# Dining with wolves: Are the rewards worth the risks?

**DOI:** 10.1371/journal.pone.0319565

**Published:** 2025-03-24

**Authors:** Summer N. Richman, Diana F. Tomback, Nels Grevstad, Darlene Kobobel

**Affiliations:** 1 Department of Integrative Biology, University of Colorado Denver, Denver, Colorado, United States of America; 2 Department of Mathematics and Statistics, Metropolitan State University of Denver, Denver, Colorado, United States of America; 3 Colorado Wolf and Wildlife Center, Divide, Colorado, United States of America; Universidade Federal de Mato Grosso do Sul, BRAZIL

## Abstract

Where wild populations of gray wolves (*Canis lupus*) occur in North America, common ravens (*Corvus corax*) and, in western regions, black-billed magpies (*Pica hudsonia*) (Family Corvidae), show up quickly at wolf kills and scavenge carcasses, often feeding near wolves. Ravens and magpies also visit wolf enclosures at gray wolf sanctuaries in Colorado, USA, and attempt to take food from wolves, but there is no information regarding how often they obtain food or are injured or killed. Working at the Colorado Wolf and Wildlife Center, Divide, Colorado, we asked whether ravens and magpies associate with gray wolves at feeding time; what proportions of ravens and magpies obtain food per enclosure; whether individual wolves react differently to the birds; and if the birds are harmed by interacting with wolves. We also examined the effects of food type, amount, and air temperature on bird numbers. We counted raven and magpie numbers in wolf enclosures and wolf and bird behaviors during daily feeding tours across 20 visits and within eight wolf enclosures per visit. Wolf reactions within each enclosure were categorized as chasing or ignoring birds or removing food. Cumulatively, across all dates and enclosures, 33% of ravens and 43% of magpies obtained food within each enclosure. Because birds were not individually marked, these percentages could be higher. Individual wolves differed in responses to ravens and magpies but most often ignored bird presence. We found no effect of food type on bird numbers but a trend in reward probability with greater food amount for ravens. There were, however, statistically significant negative relationships between daily maximum and average temperature and raven numbers, and significant positive relationships between daily minimum and average temperature and magpie numbers. We conclude that dining with wolves represented a successful foraging strategy with low risk to ravens and magpies.

## Introduction

Birds of the family Corvidae, i.e., ‘corvids,’ in general are known for highly developed cognitive abilities and social interactions (see review in [1]). Ravens and crows (*Corvus* spp.) show great adaptability to different environments [2,3]. Notably, some crows use tools to obtain food [4,5], and nutcrackers (*Nucifraga* spp.) and jays, which cache seeds and nuts for future use, have accurate spatial memory for thousands of specific locations [6–8]. Comparative studies suggest that corvid cognition is similar to that of the great apes [1,9]. Additionally, corvids exhibit play behavior, which corresponds to higher cognitive abilities [10]. These higher cognitive abilities enable corvids to locate and forage on different food types [11]. For example, the common raven (*Corvus corax*) and black-billed magpie (*Pica hudsonia*) are omnivorous and opportunistic foragers, eating fruits and berries, nuts, insects, invertebrates, bird eggs, and nestlings; and they also scavenge carcasses, seek human foods, and occasionally prey on small vertebrates [2,11–13]. Both corvid species also cache food for future use [2,11–13].

In North America, a manifestation of higher cognition and strategic foraging is that common ravens readily associate with gray wolves (*Canis lupus*), showing up within minutes at fresh wolf kills [2,14,15]. For example, in Yellowstone National Park, Stahler et al. [14] documented ravens at 100% of the wolf kills observed, which were primarily elk (*Cervus canadensis*) [16]. In the park, ravens are among the most abundant species of a scavenger community that frequents wolf kills [16,17]; and in Isle Royal National Park, ravens occurred at all wolf-killed moose (*Alces alces*) carcasses recorded over 32 years [15]. Where wolves and black-billed magpies are sympatric, magpies also appear at wolf kills, albeit in smaller numbers and less predictably [14,16]. Wolves can tear through the pelage and skin of carcasses, providing access to meat for ravens and magpies, as well as numerous other scavengers, which cannot penetrate the tough integument themselves [2,16,18].

Although the association between wolves and ravens has been observed by North American and European naturalists and scientists, this phenomenon has been historically described by many indigenous peoples across the Holarctic [2]. It is an important example of key indigenous or traditional ecological knowledge (TEK) (e.g., *sensu* [19]). For example, the Anishinaabe witnessed ravens and wolves working together to find food (S1 Text). Based on traditional knowledge, in 2021 the Ojibwe Nations of the Anishinaabe had warned the Wisconsin Department of National Resources that wolf hunting could have major impacts not just on wolves but also on the species that associate with them [20].

The reintroduction of wolves to Yellowstone National Park provided opportunities to study interactions between wolves and corvids. In the park, ravens were documented accompanying wolves during non-predatory activities, including traveling with them; and the wolf-raven relationship had some degree of exclusivity in that ravens were present in areas where wolves were active but absent in areas of coyote and elk activity [14]. Stahler et al. [14] note that because ravens have a close association with wolves, they find wolf kills rapidly. Even as wolves are pursuing prey, ravens fly overhead [14]. Anecdotal evidence also suggests that ravens may purposefully lead wolves to potential prey, such as elk or deer, and injured animals [2,14].

In the wild, wolves can lose substantial amounts from a carcass each day to raven scavenging [21,22], which is a disadvantage to wolves from this association. Stahler et al. [14] observed from 3 to 135 ravens, with a mean of 28.6 ravens, at wolf-killed carcasses, indicating potentially large losses to ravens. Ravens and magpies, however, also risk injury or death from aggressive wolves at carcasses [14,21]. Despite the disadvantages and risks, some researchers speculate there is a unique co-evolutionary relationship between wolves and ravens [2,3,14]. Magpies, on the other hand, may be opportunistic scavengers with little potential reciprocity with wolves.

Interactions among ravens, magpies, and wolves have also been observed at wildlife and wolf sanctuaries. For example, caretakers at a wolf sanctuary in Austria report the presence of ravens and Eurasian magpies (*Pica pica*) in wolf enclosures [23]. Similarly, we (DFT and DK) have witnessed ravens and black-billed magpies associating with wolves at two wolf sanctuaries in Colorado, and we have reports of both corvids routinely present at a third sanctuary in the state (S2 Text). Sanctuaries in Colorado report ravens occasionally killed by wolves in their enclosures. However, in contrast to studies in the wild indicating that ravens and magpies often obtain food at wolf-killed carcasses, no information has been available on the nature of incentives or rewards to ravens and magpies interacting with wolves at sanctuaries that would justify the risk.

Given that wolves have been extirpated from many regions within the western U.S. for nearly a century, and the last wild wolf was killed in Colorado in 1945 [24], spontaneous associations at wolf sanctuaries and even in the wild suggest that interactions between ravens and wolves are potentially instinctive, likely go beyond simple opportunism, and may result from a long coevolutionary history reinforced by cultural transmission [2,14]. In other words, associating with wolves appears adaptive for ravens because it could provide access to food under different circumstances, and wolf attention to raven cues could lead them to potential prey [14,15].

We studied the feeding interactions among gray wolves, common ravens, and black-billed magpies at the Colorado Wolf and Wildlife Center (CWWC) in Divide, Colorado. Since 1993, the Center has served as a regional educational institution and sanctuary for wolves. The CWWC is unusual among wolf sanctuaries, because pairs of wolves are housed in large, fenced enclosures.

Our goals for this study were the following: To determine (1) whether ravens and magpies (the ‘corvids’) frequent wolf enclosures at the CWWC when wolves are fed; (2) whether ravens or magpies or both obtain food from wolf enclosures and what proportions of birds are successful within each enclosure; and (3) whether there are predictive correlates of daily corvid numbers and how these vary by wolf enclosure. We also determined whether individual wolves differ in their reactions to the corvids, if the corvids are at risk by interacting with wolves, and whether the relationship with other captive canids at CWWC is similar.

## Methods

### Study location and site description

The CWWC is an Association of Zoos and Aquariums (AZA) accredited facility in Divide, Colorado. The CWWC is dedicated to educating the public and school groups about wolf behavior, ecology, and especially wolf conservation. The CWWC houses wolves that come from zoos, sanctuaries, and facilities which breed wolves for educational purposes. The CWWC supports the U.S. Fish and Wildlife Service (USFWS) by offering refuge to critically endangered species and subspecies, such as American red wolves (*Canis rufus*) and Mexican gray wolves (*Canis lupus baileyi*). Other gray wolf subspecies at the CWWC include arctic wolves (*Canis lupus arctos*) and British Columbian wolves (*Canis lupus columbianus*). In addition, the CWWC houses other canid species for educational and conservation purposes, such as coyotes (*Canis latrans*), red foxes *(Vulpes vulpes)*, swift foxes *(Vulpes velox*), and New Guinea singing dogs (*Canis lupus dingo or Canis hallstromi* but see [25]).

**Fig 1:**
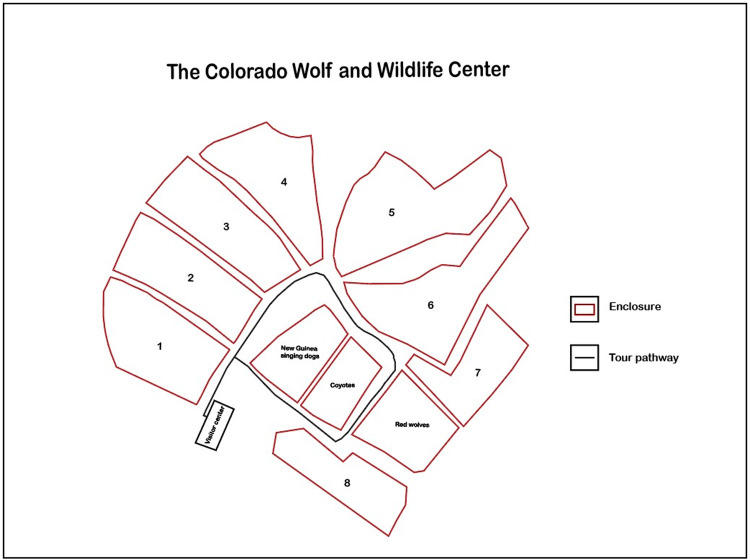
Layout of the CWWC, including wolf, coyote, and New Guinea singing dog enclosures. The feeding tour pathway is indicated on the map. The numbers indicate wolf enclosure number.

The CWWC was built within a natural, open ponderosa pine (*Pinus ponderosa*) forest at about 2,797 m a.s.l. (9,175 ft) in elevation. The 6-hectare (15-acre) facility is surrounded by a perimeter fence, which serves as an additional barrier to keep resident animals in and prevents wildlife and vandals from entering the premises. Out of ten enclosures total, there are eight gray wolf enclosures in proximity, each surrounded by a 2.4-m (8 ft) high fence and with a single double-door vestibule entrance. Each enclosure ranges from 0.6 to 0.8 ha (1.5 acres to ~ 2 acres) in area (Fig 1). All except two enclosures have high tensile game fencing. The American red wolf and Mexican gray wolf enclosures have chain link fences, per USFWS protocol. The enclosures are all within forested habitat on hilly terrain, with 1 to 2 wolves per enclosure. A total of 14 wolves in the eight gray wolf enclosures routinely provided data for this study (Table 1). The enclosures are accessed by a single walking path that leads from the main entrance through the CWWC. This path is used by all visitors and wolf caretakers.

**Table 1:** Wolves observed in food interactions with ravens and magpies at the Colorado Wolf and Wildlife Center.

Wolf I.D. No.	Enclosure	Age	Sex	Origin	Age at Arrival
1-1	1	8	M	Breeding facility (TX)	5 wk
1-2	1	8	F	Zoo (FL)	3 yr
2-1	2	8	M	Zoo (FL)	3 yr
2-2	2	13	F	Zoo (Columbia)	7 wk
3-1	3	5	M	Breeding facility (TX)	5 wk
3-2	3	4	F	Zoo (FL)	5 wk
4-1	4	5	M	Breeding facility (TX)	6 mo
4-2	4	4	F	Zoo (FL)	5 mo
5-1	5	12	M	Zoo (FL)	6 wk
6-1	6	12	M	Breeding facility (TX)	5 wk
6-2	6	11	F	Born at the CWWC	N/A
7-1	7	4	M	Wolf sanctuary (CA)	1 yr
7–2	7	4	M	Wolf sanctuary (CA)	1 yr
8-1	8	14	M	Zoo (FL)	5 wk

Wolf identification number, enclosure number, age (years) at the start of data collection, sex (M =  male, F =  female), origin (TX =  Texas, FL =  Florida, CA =  California), and age on arrival at CWWC (yr =  year, mo =  months, wk =  weeks).

### Wolf identification and enclosures

During our study, a few wolves were moved to different enclosures for various reasons. To maintain consistency, we assigned each wolf an identification number based on its enclosure number at the start of the study, followed by a hyphen and a 1 for male and 2 for female to differentiate wolves in a pair. The only exception to the rule are the Mexican gray wolves, 7-1 and 7-2, which are both male. During daily feeding tours, wolves are fed in consecutive order from enclosure 1 to 8. There are five enclosures with male-female pairs, one enclosure, 7, with two male wolves, and two enclosures, 5 and 8, which each housed a single male wolf (Table 1). After the study began, wolf 2-1 and wolf 2-2 were relocated to enclosure 8, which was formerly wolf 8-1’s enclosure, and four wolf pups were placed in enclosure 2. Observations associated with the pups in 2 are not included in the study, given that they attracted higher numbers of corvids compared to the adult wolf enclosures.

All enclosures include a water source for the wolves and many trees, which serve as perches for visiting corvids. The Mexican gray wolves, housed in enclosure 7, have less contact with humans than the other wolves at the CWWC. For example, staff minimize interactions with the Mexican gray wolves, and they do not schedule visitation (small group interactions) in enclosures with this subspecies. The public, however, views them from a distance during CWWC tours.

The wolves differ somewhat in background (Table 1). Seven of the wolves came from a zoo in Florida, four of them came from a breeding facility in Texas, and two from a wolf sanctuary in California. In an unusual circumstance, wolf 2-2 became pregnant at the age of 1 year while at the CWWC. Most females attain reproductive maturity at 2 to 4 years of age [26]. The pup born to wolf 2-2 became wolf 6-2. The two Mexican gray wolves, 7-1 and 7-2, were living in a wolf sanctuary in California when the USFWS brought them to the CWWC. At the time, they were both 1 year old. The two Mexican gray wolves are litter mates.

### Study protocol

We visited the CWWC on 20 different days between 29 October 2021 and 21 October 2022, avoiding inclement or extreme weather, for about 4 hours per visit. We start observing wolves and corvids about 2 hours before and then during the evening “feeding tour,” which starts at 4 pm during winter hours (September 10^th^ through May 13^th^) and 6 pm during summer hours (May 14^th^ through September 9^th^). Each tour lasts 60 to 75 minutes. The tour is led by two knowledgeable and experienced staff members who are typically accompanied by 20 to 25 visitors but as many as 40. The guests are generally quiet and attentive or ask questions as the tour leaders lecture and then stop in front of each enclosure and provide background on each wolf or wolf pair in the enclosure. At each stop, the leaders throw chunks of meat, fowl, or fish over the fence. Wolves and the other animals at the CWWC are fed a variety of donated foods, but the wolves all receive the same food type and roughly the same food amount during a feeding tour.

During our study, the tour and our observations always began at enclosure 1, located near the main entrance (Fig 1). We follow behind the tour, stopping at each enclosure in turn to observe and document corvid-wolf interactions. Most often the wolves came down to the fence along the pathway in anticipation of being fed. The process of feeding the wolves was slow, because there were questions and discussion. Food was dispensed by one of the tour leaders, who threw pre-measured portions of food over the fence closest to the pathway, aiming at a spot near the wolves. Standing in front of each enclosure, we recorded wolf and corvid behaviors using an iPhone 13 Pro. Data were usually entered directly into a spreadsheet with an iPad Pro (Apple, 3^rd^ generation). Interactions between the wolves and birds were observed in real time, and key moments were reviewed by watching the videos while at the CWWC or later for transcribing additional data.

The time of each wolf feeding was recorded, along with the number of ravens and magpies in each enclosure. At every enclosure, we noted when a raven or a magpie obtained food, and how each wolf or wolf pair responded to each bird present. We categorized wolf reactions as follows: ignore a bird, chase a bird, or snatch food before it is taken by a bird. We noted the number and category of individual wolf reactions. We determined the proportion of ravens and magpies rewarded in each enclosure and across all visits. Because there was potential for a wolf to react differently to individual birds, we counted wolf reactions in response to all birds in each enclosure. The number of wolf reactions documented for each enclosure was equal to the number of corvids in that enclosure.

As a provision of its accreditation through the AZA, the CWWC is encouraged to do research at their facility, and our work was considered to be under their jurisdiction. We were not required by our research administration to develop an IACUC protocol for this study, as confirmed by Laura Richardson, CPIA Administrator, Office of Research Committee Support, University of Colorado Denver |  Anschutz Medical Campus. In a letter dated February 9, 2022, she stated: “If the wolves are just being observed and not being interfered with or manipulated, an IACUC protocol is not needed unless the funding agency has requested it.” An IACUC protocol was not requested by the undergraduate research funding program (EURēCA!) at the University of Colorado Denver.

### Predicting numbers of ravens and magpies over time

The number of ravens and magpies that visited the wolf enclosures varied among our visits and with time of year. We asked whether differences in raven and/or magpie visitation could be related to differences in food type or food amount fed to each wolf, or differences in daily minimum, maximum, or average temperature (°C), which could influence the caloric requirements of the birds. Minimum and maximum daily temperatures in Divide, CO were acquired using the AccuWeather website, https://www.accuweather.com.

Daily food types and amounts were obtained from food logs kept by the CWWC. The CWWC began keeping food logs on February 26, 2022, so we do not have food type data for earlier visits. Data from the food log included: date, enclosure number, enclosure species, individual animal, gender, time of feeding, food amount (kg), food type, and staff member’s name. The main food types fed the wolves were chicken, turkey, beef, and elk. Wolves require more energy in the colder weather, so the CWWC provided a greater amount of food during winter.

### Statistical analyses

To examine how many birds of each species a wolf feeding typically attracted, what proportion of them were typically rewarded, how much those numbers varied across feedings, and whether they differed across enclosures, we computed for each enclosure their means and standard deviations (sd) over the 20 visitation dates. We also computed the same summary statistics for all eight enclosures combined, with a 95% confidence interval (Wilson score type) for the overall proportion rewarded.

In separate analyses, we investigated whether food type predicted the presence of ravens or magpies but focused on the four most frequently fed food types. We classified each wolf feeding according to whether a given food type was provided (yes/no) and whether ravens or magpies were present (presence/absence). This produced for each corvid species four two-by-two contingency tables, one for each main food type, indicating corvid presence and absence rates when a given food was provided and when it was not. Then for each table we performed a chi-squared test to determine if corvid presence depended on whether the food was provided. We checked the large sample size assumption for each test using the well-established criterion that the expected cell counts (under the null hypothesis) should all be five or larger.

To investigate relationships between numbers of corvids and continuous variables (temperature and food amount), we carried out a mixed-effects zero-inflated Poisson regression analysis (after determining the corvid counts were zero-inflated using the method described in [27]). In this model, a count is either a Poisson variable with expected value µ  or it is one of the excess zeros, the probability of which is *p* [27]. The parameter µ  is allowed to depend on predictor variables. We let µ  depend on enclosure, food amount, temperature, and visitation date via the model


$$Log\left({µ}_{ij}\right)=\;\beta_{0i}+\;\beta_{1}\;\times \;X_{1ij}+\;\beta_{2}\;\times \;X_{2j}+\;\alpha_{j}$$ (1)

where µ_*ij*_ is the expected number of corvids of a given species (ravens or magpies) in the *i*th enclosure (*i* =  1, 2, …, 8) on the *j*th visitation date (*j* =  1, 2, …, 20), β_0*i*_ is an enclosure-specific intercept representing the effect of the *i*th enclosure (for example due to corvid preferences for certain enclosures over others), *X*_1*ij*_ is the food amount (kg) in the *i*th enclosure on the *j*th visitation, and *X*_2*j*_ is the temperature (°C) on the day of the *j*th visitation. Daily high, daily low, and the average of daily high and low temperatures were all investigated as potential predictors. Enclosure, food amount, and temperature are all fixed effects in the model. The term α_*j*_ is a random effect of the *j*th visitation date, common to all enclosures. This random effect term is used to model spatial correlation in corvid counts across enclosures during a given visitation—that is, covariation in the counts due to spatial proximity of the enclosures. Such covariation could reflect, for example, fluctuations (across visitation dates) in corvid densities in the vicinity of the wolf sanctuary and corvid movement between enclosures during the wolf feedings on a given date. The slope coefficients β_1_ and β_2_ quantify the expected change in the *log* number of corvids present for each one-unit change in food amount or temperature. Thus, *e*^β1^ and *e*^β2^ are the expected *multiplicative* changes in µ  and are called incident rate ratios [27]. The coefficients β_0*i*_, β_1_, and β_2_ and the excess-zero probability *p* were estimated from the data by the maximum likelihood method. Statistical significance of the estimated coefficients was assessed using Wald *Z* tests with significance level at 0.05.

We also tested for a relationship between the probability of a bird obtaining food and daily temperature or food amount by carrying out (separately for ravens and magpies) logistic regression analyses, where the dichotomous response variable for each corvid present during a given wolf feeding was whether the bird obtained food or not, and the predictors were enclosure, temperature, and food amount. Thus, the model was


$$Log\left(O_{ij}\right)=\beta_{0i}+\beta_{1}\times X_{1ij}+\beta_{2}\times X_{2j}$$ (2)

where *O*_*ij*_ is the odds of an individual corvid in the *i*th enclosure obtaining food during the feeding on the *j*th visitation date, β_0*i*_ is an enclosure-specific intercept representing the effect of the *i*th enclosure on the likelihood of the corvid obtaining food, and *X*_1*ij*_ and *X*_2*j*_ are again the food amount (kg) and temperature (C). Daily high, low, and average temperatures were all investigated as potential predictors. Model coefficients were again estimated by maximum likelihood and statistical significance assessed using Wald *Z* tests at the 0.05 level.

We used R statistical software (version 4.2.2) for all data analysis. We performed the zero-inflated Poisson regression analyses using the ‘glmmTMB’ R package [28].

## Results

### Corvid behavior during feeding tours

Ravens were present during every visit we made to the CWWC, but on some dates magpies were not present, which resulted in many counts of zero. Typically, about 30 minutes before the feeding tour began, corvids would start arriving at the CWWC, flying in from different directions. The first corvids to show up would fly over enclosures and vocalize. At the start of a feeding tour, as the wolf caretaker carrying a container of food approached the first wolf enclosure, ravens and magpies would fly to the enclosure, landing on the ground near the wolves or perching near the wolves in trees or on the fence that borders the enclosure. The caretaker would throw chunks of food over the fence near the wolves.

**Fig 2:**
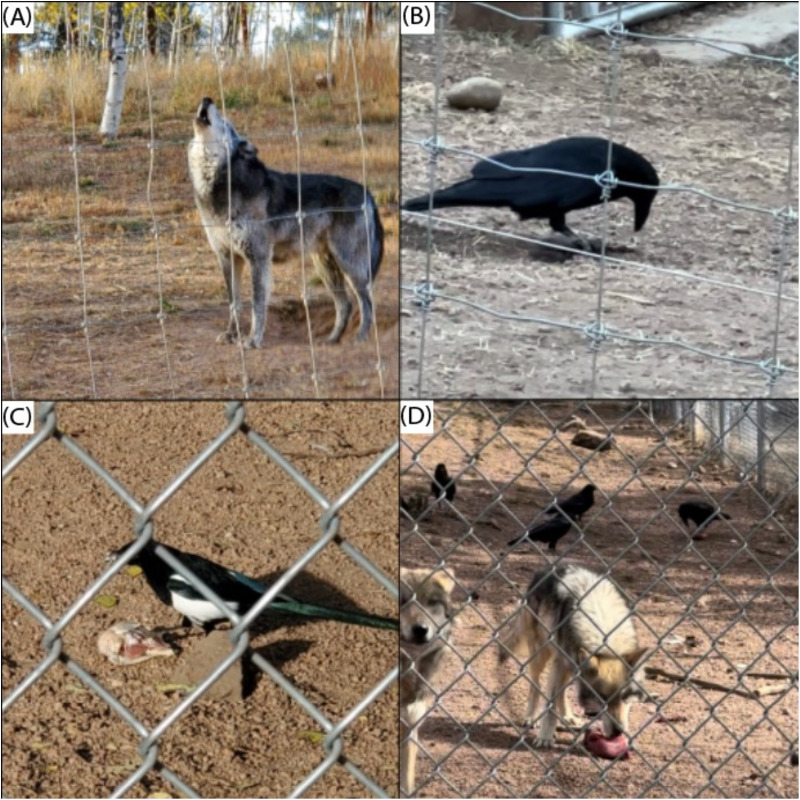
(A) Gray wolf 6-1 howling during an afternoon feeding tour (photo credit: D.F. Tomback), (B) Common raven eating food provided to wolves during a feeding tour (photo credit: S.N. Richman), (C) Black-billed magpie taking food provided to wolves during the feeding tour (photo credit: D.F. Tomback), (D) Ravens and Mexican gray wolves feeding on food provided during the feeding tour (photo credit: S.N. Richman).

After food was tossed into the enclosures, ravens and magpies usually differed in their choice of perch. Ravens initially perched in trees or on the ground farther from the wolves and their food than magpies. Magpies typically moved to the ground close to the food. After a short time with food present, some of the ravens high in the trees would fly down to lower branches or move to the ground. Both corvid species would walk up to unguarded food chunks and either peck at them in place or fly off with a piece or the entire chunk (Fig 2B–2D).

The ravens appeared more cautious than magpies around the wolves and wary of the people on the public feeding tour but less concerned about our presence. Some of the ravens would not land on the ground until the tour crowd moved to the next enclosure. As the tour approached the next enclosure, some birds remained in the previous enclosure, while others moved to the next enclosure. The corvids typically followed the order of enclosures with the feeding tour, but some birds lagged by 1 or 2 enclosures. At the end of each feeding tour, the tour leader initiated a howling chorus of wolves and visitors, and the corvids appeared unperturbed by the sound (Fig 2A). On some observation days, after the feeding tour, ravens and magpies remained in enclosures into the evening after the CWWC closed. On other days, nearly all the birds left within 10 minutes after the conclusion of the feeding tour.

### Numbers of ravens and magpies and proportions obtaining food

**Fig 3:**
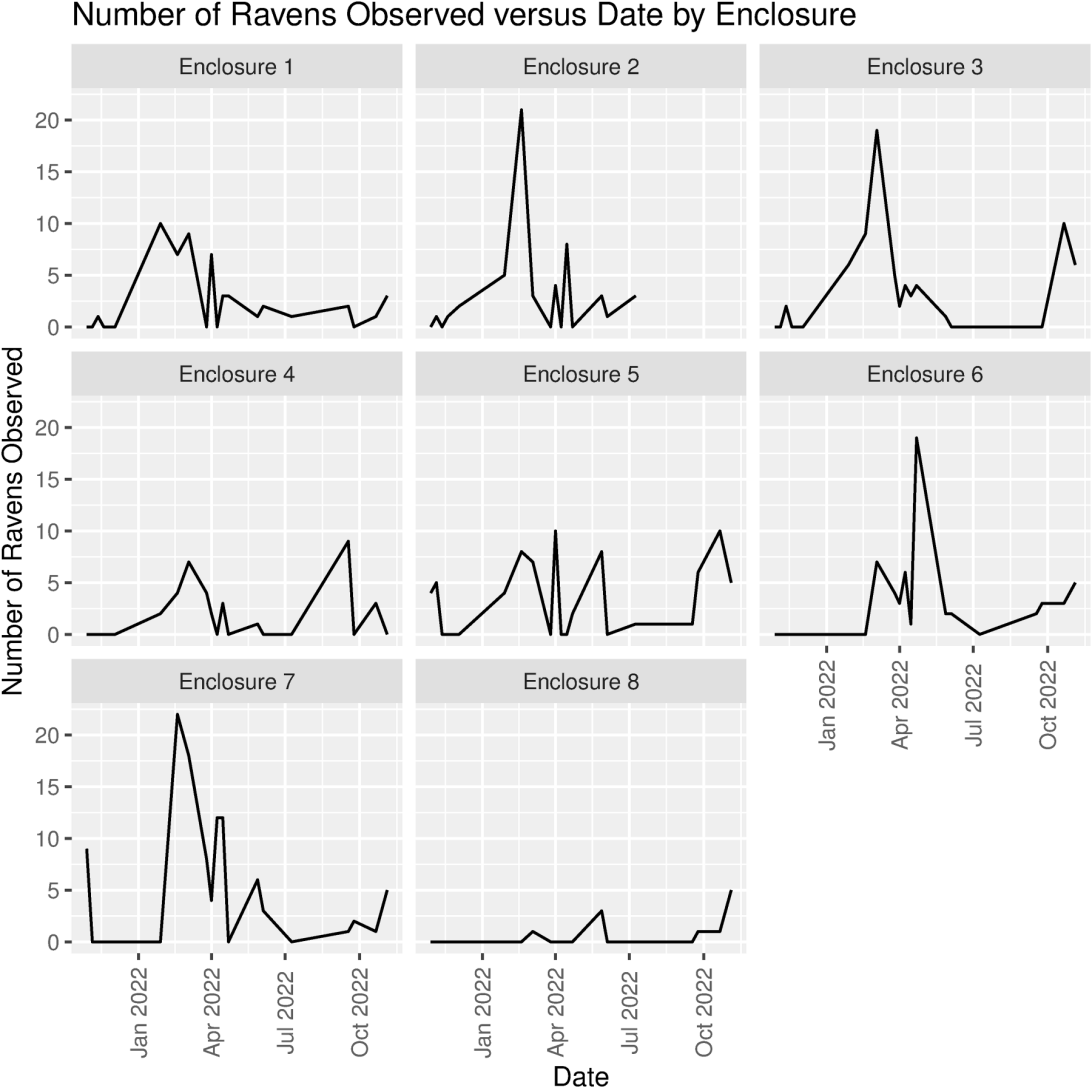
Number of ravens observed during wolf feedings versus visitation date by enclosure.

The number of ravens varied both among enclosures and among visits (Table 2 and Fig 3). The number of ravens per enclosure during a wolf feeding ranged from 0 to 22. The highest number of ravens observed in an enclosure across all dates, 22 ravens, was in enclosure 7, which houses the Mexican gray wolves. On average (across all enclosures and visits), a wolf feeding attracted 2.9 ±  4.3 (sd) ravens in each enclosure, with enclosure 7 attracting the highest mean of 5.2 ±  6.5 ravens and enclosure 8 the lowest mean of 0.6 ±  1.3 (Table 2).

**Table 2:** Daily mean and cumulative numbers of birds observed per enclosure and proportion that obtained food.

Encl.	Ravens			Magpies		
	Mean (sd) number observed	Mean (sd) proportion rewarded	Cumulative proportion rewarded	Mean (sd) number observed	Mean (sd) proportion rewarded	Cumulative proportion rewarded
1	2.5 (3.2)	0.55 (0.42)	0.50 (25/50)	0.5 (1.6)	0.31 (0.27)	0.40 (4/10)
2	3.2 (5.2)	0.28 (0.38)	0.21 (11/52)	0.4 (0.9)	0.17 (0.33)	0.29 (2/7)
3	3.5 (4.8)	0.14 (0.20)	0.17 (12/71)	0.7 (1.3)	0.23 (0.32)	0.31 (4/13)
4	1.8 (2.6)	0.08 (0.13)	0.14 (5/35)	0.3 (0.8)	0.33 (0.58)	0.33 (2/6)
5	3.5 (3.6)	0.33 (0.27)	0.38 (27/71)	0.7 (1.2)	0.50 (0.45)	0.54 (7/13)
6	2.9 (4.4)	0.32 (0.35)	0.44 (25/57)	0.3 (0.6)	0.25 (0.42)	0.29 (2/7)
7	5.2 (6.5)	0.28 (0.30)	0.34 (35/103)	0.4 (0.8)	0.70 (0.45)	0.78 (7/9)
8	0.6 (1.3)	0.68 (0.46)	0.64 (7/11)	0.0 (0.0)	NA (NA)	NA (0/0)
All	2.9 (4.3)	0.32 (0.35)	0.33 (147/450)	0.4 (1.0)	0.37 (0.41)	0.43 (28/65)

Means and standard deviations in parentheses (sd) of daily counts of birds observed and proportions that obtained food ( = rewarded) during feedings, by enclosure, across 20 visits to the CWWC. Also shown for each enclosure is the cumulative proportion of birds that obtained food over the 20 visits (ratio of cumulative number rewarded to cumulative number observed, as shown in parentheses). No magpies were observed in enclosure 8. The bottom row shows the same statistics but computed from all 160 ( = 8 × 20) enclosure and visitation date combinations.

**Fig 4:**
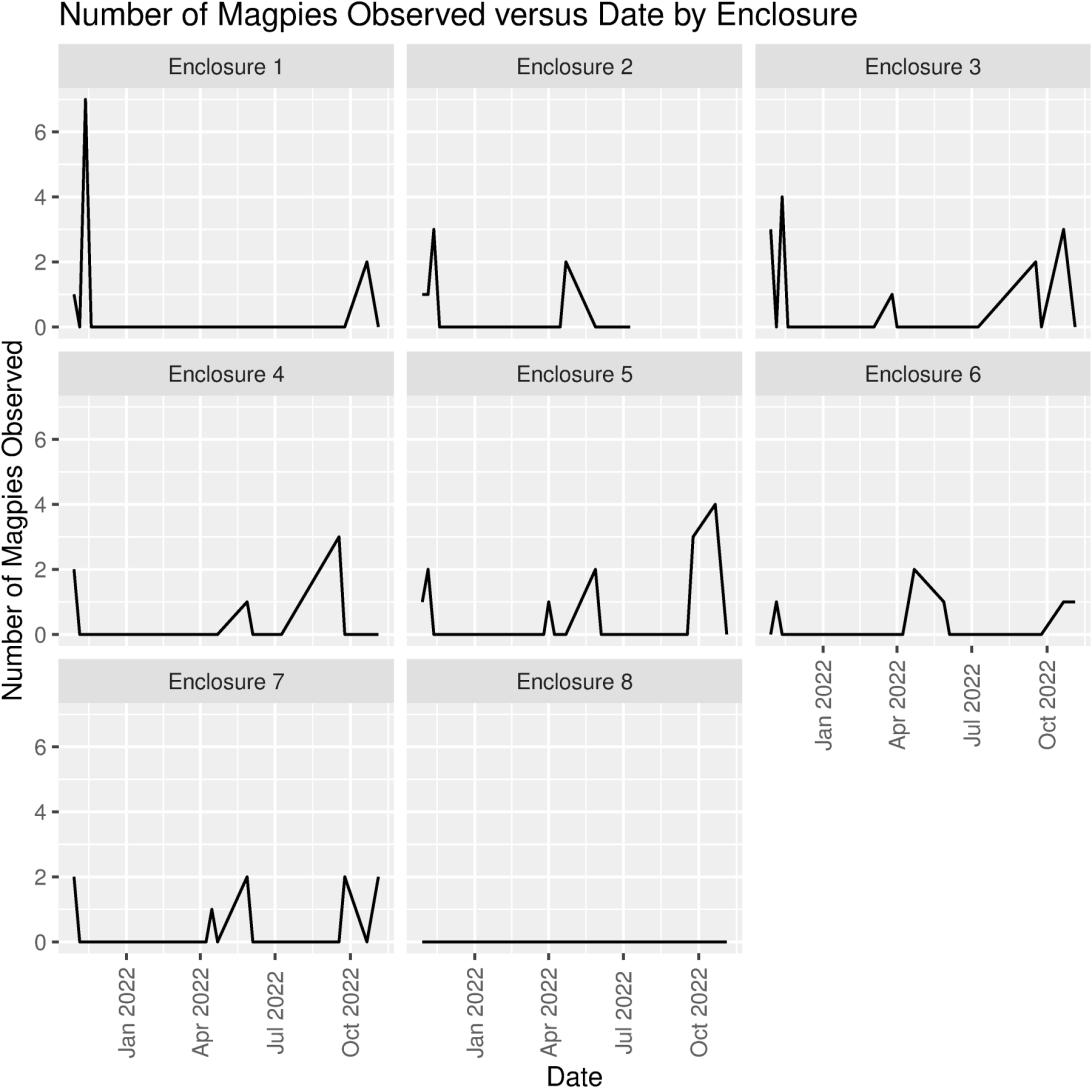
Number of magpies observed during wolf feedings versus visitation date by enclosure.

Many fewer magpies than ravens routinely visited wolf enclosures during wolf feedings. The number observed per enclosure ranged from 0 to 7 and did not vary as much across enclosures and visits as did the number of ravens (Table 2 and Fig 4). On average (across all enclosures and visitations), only 0.4 ±  1.0 (sd) magpie per enclosure was attracted to a wolf feeding, with small differences in mean numbers of magpies among enclosures (Table 2). No magpies were observed in enclosure 8 during any of the 20 visits to the sanctuary.

Magpies were slightly more successful than common ravens in obtaining food from wolf enclosures, despite their small numbers. Across enclosures, the average proportion of ravens that obtained food was 0.32 ±  0.35 (sd); and the average proportion of magpies that obtained food was 0.37 ±  0.41 (sd). Cumulatively over the study, 0.33 of ravens (with 95% CI 0.28 to 0.36) and 0.43 of magpies (with 95% CI 0.31 to 0.56) obtained food within each enclosure (Table 2). The proportion of ravens obtaining food per enclosure ranged from 0.14 to 0.64. The highest proportion of ravens obtaining food was in enclosure 8, and the lowest proportion rewarded was in enclosure 4. The proportions of magpies obtaining food per enclosure ranged from 0.29 to 0.78 with the highest proportion obtaining food from enclosure 7 and the lowest proportion from enclosure 2 (Table 2). Given that corvids were not marked for this study, it is possible that individual ravens and magpies were more successful in obtaining food than observed because they visited different enclosures. In other words, a bird that did not obtain food in one enclosure could have obtained food in another enclosure.

### Wolf behavior towards ravens and magpies

The interactions that we observed between wolves and corvids were generally benign. Given a cumulative total of 450 ravens across all dates within all enclosures, we observed wolves chase only 6 ravens, take food away from only 1 raven, and ignore 443 ravens. Of the 6 ravens chased over the entire study, 2 were in each of enclosures 1, 2, and 3. Wolves 1-1, 2-1, and 3-2 chased the ravens. On one occasion only, in enclosure 2, a raven’s access to food was thwarted by a wolf, which took the food chunk away. Out of the cumulative total of 65 magpies that we observed over the study, we observed 2 magpies chased and 63 ignored. The two magpies chased were both in enclosure 3, and there were none chased in any of the other enclosures. On two occasions, once in enclosure 1 and once in enclosure 3, a wolf took food away from a magpie.

Thus, during our observations, we rarely observed wolves chase ravens and magpies from food. The birds appeared to be tolerated within the enclosures. We never observed a wolf harm a raven or magpie. However, staff members at CWWC reported that ravens and magpies were occasionally killed by resident wolves during the past several years. For example, prior to the start of this study, wolf 3-1 caught and killed a raven near its food. During our study but on a day we were not present, wolf 2-1 killed a magpie and then reportedly “played with it” by throwing the bird about.

### Minimum and maximum temperature and food amount as predictors of bird numbers

Given the variation in raven and magpie numbers across enclosures and visitation dates, we asked whether daily temperature, food amount fed to each wolf, or food type predicted trends in raven and magpie occurrence. The zero-inflated Poisson regression analysis indicated that the number of ravens present during a given wolf feeding tour had a statistically significant negative relationship with daily high (β =  -0.046, Std. Err. 0.020, p =  0.019) and daily average (β =  -0.048, Std. Err. 0.023, p =  0.037) temperatures but not with daily low temperature (Table 3). Results also indicated that the number of ravens present during a wolf feeding was not significantly related to food amount provided in each enclosure (Table 3).

**Table 3:** Number of ravens and magpies present versus temperature and food amount.

M	Predictor	Ravens				Magpies			
		β Est.	Std. Err.	*Z*	p-value	β Est.	Std. Err.	*Z*	p-value
1	Daily High Temp.	-0.046	0.020	-2.34	**0.019**	0.065	0.046	1.42	0.155
	Food Amount	-0.076	0.118	-0.64	0.520	0.271	0.622	0.44	0.664
2	Daily Low Temp.	-0.042	0.026	-1.61	0.108	0.113	0.045	2.50	**0.013**
	Food Amount	-0.074	0.119	-0.62	0.535	0.423	0.537	0.79	0.431
3	Daily Avg. Temp.	-0.048	0.023	-2.09	**0.037**	0.095	0.044	2.15	**0.032**
	Food Amount	-0.074	0.118	-0.63	0.530	0.427	0.565	0.76	0.449

Results of mixed-effects zero-inflated Poisson regression analyses, for each corvid species, where the response variable is number of corvids present during a wolf feeding and the predictors are temperature (daily high, low, or average for models (M) 1, 2, and 3, respectively) and food amount. Shown for each model and each predictor are the estimated coefficient β, its standard error, and the test statistic (*Z*) and p-value for the test of the null hypothesis that the true β is 0. Bold font indicates p-value <  0.05. The enclosure-specific estimated intercepts (not shown) ranged from 0.32 to 3.52 for ravens and from -28.06 to -1.74 for magpies.

**Fig 5:**
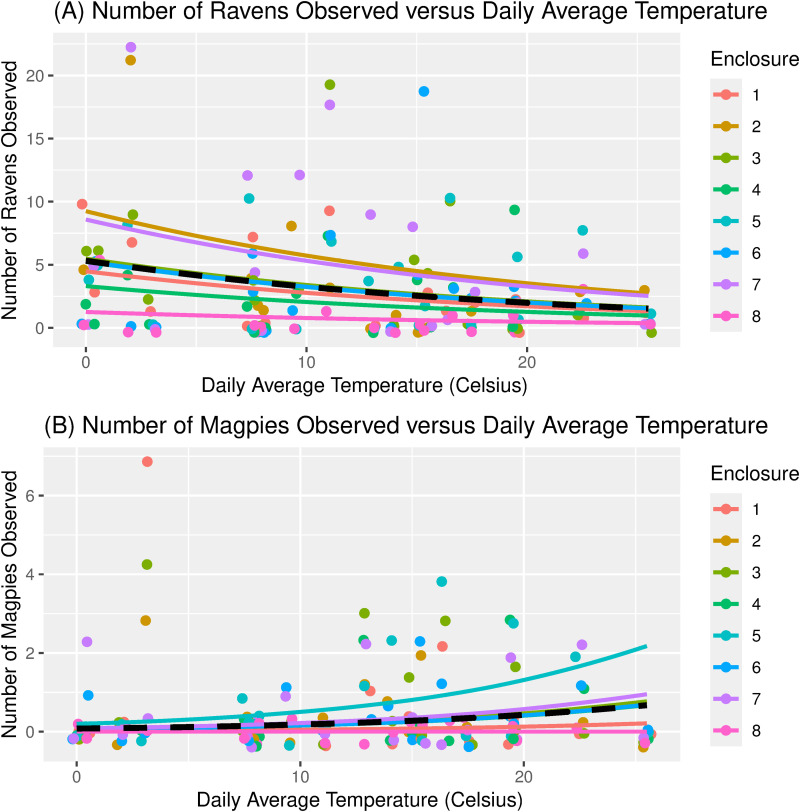
(A) Number of ravens and (B) number of magpies observed at wolf feedings versus daily average temperature. In both graphs, the solid curves correspond to the fitted zero-inflated Poisson regression model. The black dashed curve is an average of the enclosure-specific curves.

The negative slope coefficients β =  -0.046 for high temperature and β =  -0.048 for average temperature indicated that each additional 1°C increase in temperature led to a 4.5% and 4.3% decrease, respectively, in the expected number of ravens (*e*^-0.046^ =  0.955 and *e*^-0.048^ =  0.957) (Fig 5A). The negative slope coefficients for food amount suggested that fewer ravens were present when more food was available per enclosure, but the relationship was not statistically significant (Table 3).

Temperature also predicted the number of magpies present, but it was a positive relationship (the reverse of the relationship for ravens). More specifically, the Poisson regression analyses indicated that daily low (β =  0.113, Std. Err. =  0.045, p =  0.013) and daily average (β =  0.095, Std. Err. =  0.044, p =  0.032) temperatures, but not daily high temperatures, predicted the number of magpies present during a wolf feeding (Table 3). The positive slope coefficients β =  0.113 and β =  0.095 for daily low and daily average temperatures indicate that each additional 1°C increase in temperature led to a 12% and 10% increase, respectively, in the expected number of magpies (*e*^0.113^ =  1.12 and *e*^0.095^ =  1.10) (Fig 4B). The positive slope coefficients for food amount suggested that higher numbers of magpies were present when more food was available per enclosure, but the relationship was not statistically significant (Table 3).

The logistic regression analysis indicated a positive relationship between the probability that a raven will obtain food and the amount of food provided in the enclosure during a wolf feeding, suggesting that more available food increases the chance of a raven obtaining food, but the relationship indicated a trend and was not statistically significant (Table 4). In addition, the probability of a raven obtaining food was not statistically significantly related to temperature (Table 4).

**Table 4:** Probability of corvids being rewarded versus temperature and food amount.

M	Predictor	Ravens				Magpies			
		β Est.	Std. Err.	*Z*	p-value	β Est.	Std. Err.	*Z*	p-value
1	Daily High Temp.	0.015	0.015	1.00	0.319	0.53	0.363	1.45	0.147
	Food Amount	0.358	0.184	1.94	0.052	5.744	4.502	1.28	0.202
2	Daily Low Temp.	0.005	0.018	0.31	0.767	0.237	0.163	1.46	0.144
	Food Amount	0.351	0.183	1.92	0.055	2.457	2.016	1.22	0.223
3	Daily Avg. Temp.	0.012	0.017	0.71	0.486	0.575	0.391	1.47	0.142
	Food Amount	0.354	0.184	1.93	0.054	6.225	4.707	1.32	0.186

Results of logistic regression analyses, for each corvid species, where the dichotomous response variable for each corvid present during a feeding is whether the bird obtained food or not and the predictors are temperature (daily high, low, or average for models (M) 1, 2, and 3, respectively) and food amount. Shown for each model and each predictor are the estimated coefficient β (β Est.), its standard error (Std. Err.), and the test statistic (*Z*) and p-value for the test of the null hypothesis that the true β is 0. The enclosure-specific estimated intercepts (not shown) ranged from -2.98 to -0.41 for ravens and from -49.36 to -7.28 for magpies.

The logistic regression model indicated a positive relationship between the probability of a magpie obtaining food and food amount, but the relationship was not statistically significant (Table 4). The probability of a magpie being rewarded was also not statistically significantly related to temperature (Table 4).

### Food type

We found no relationship between the type of food provided at a feeding and corvid presence. More specifically, the chi-squared tests indicated no significant differences for any of the four main food types in corvid presence rates between feedings when the food type was provided and feedings when it was not available (Table 5). The large sample size assumption required by the chi-squared tests was met in all cases except one (magpie presence versus turkey as the food provided).

**Table 5:** Corvid presence rates for different food types and chi-squared test results.

		Ravens		Magpies	
Food type	Provided during feeding?	Proportion of feedings with ravens present	Chi-squared test results	Proportion of feedings with magpies present	Chi-squared test results
Beef	Yes	0.64	*Χ*^2^ = 0.13 p = 0.718	0.12	*Χ*^2^ = 2.38 p = 0.123
	No	0.69		0.24	
Turkey	Yes	0.53	*Χ*^2^ = 0.87 p = 0.351	0.18	*Χ*^2^ = 0.00 p = 1.000
	No	0.68		0.16	
Elk	Yes	0.60	*Χ*^2^ = 0.41 p = 0.525	0.13	*Χ*^2^ = 0.10 p = 0.757
	No	0.69		0.18	
Chicken	Yes	0.67	*Χ*^2^ = 0.05 p = 0.822	0.14	*Χ*^2^ = 0.82 p = 0.364
	No	0.63		0.23	

Proportions of wolf feedings for which corvids were present when a food type was provided and proportions for which they were present when it was not provided, reported separately for ravens and magpies. Also shown are results of chi-squared tests (test statistic *Χ*^2^ and p-value) to determine, for each food type, if the two proportions differed statistically significantly. The null hypothesis is that the two proportions do not differ.

## Discussion

Ravens and magpies frequent wolf kills in the wild. Ravens have been observed feeding routinely at carcasses in close proximity to wolves in Yellowstone and Isle Royale National Parks [14,15]. Ravens and magpies in the wild apparently risk injury or death from wolves [14,21], but the risk may be minimal, given protection by the numbers of birds present and the mobility of the birds. Vucetich et al. [15] note that wolves frequently chase ravens from carcasses. In Yellowstone National Park, Stahler et al. [14] observed a mean of 28.6 ravens and up to 135 ravens at a single carcass, and in Isle Royal National Park, Vucetich et al. [15] reported 5 to 15 ravens per moose kill.

The observations in the wild indicate that both ravens and magpies dependably obtain a high-quality food reward from feeding on wolf kills and in turn experience low risk of harm, although the amount of food they obtain collectively can be significant. Summarizing the literature, Vucetich et al. [15] suggest that ravens can take 2 to 20 kg of meat per day from a wolf-killed carcass. They suggest that the inevitable presence of scavenging ravens has driven the social system of wolves, and that larger wolf packs are able to obtain relatively more from a carcass than smaller packs. Heinrich [2] proposes that ravens select wolf-killed carcasses, depending on wolves to rip open carcasses for them, and wolf-killed carcasses also provide ravens assurance about the safety of the carcass, given attempts by humans to eradicate wolves and other wildlife regarded as destructive. Ravens are otherwise hesitant to feed on carcasses they encounter in the absence of wolves [2,14].

Ravens and magpies have also been observed to visit wolf enclosures routinely at wolf and wildlife sanctuaries in both Europe and the United States [e.g., 23]. The circumstances and dynamics of wolves in captivity differ from wolves in the wild--especially the absence of large wolf-killed carcasses. Wolves in captivity may also behave differently, given they have certainty of receiving food and are not living in packs or defending home ranges. The objectives of our study at the CWWC were to determine whether ravens and magpies enter wolf enclosures when wolves are fed, whether birds obtain food within enclosures, whether individual wolves differ in their reactions to the birds, and whether there is risk of wolf attack for the birds. We found that corvids do visit wolf enclosures and their numbers varied by visit. We examined whether there were predictive correlates of numbers. We also explored whether the relationship with other captive canids at CWWC is similar for ravens and magpies, or whether tolerance is lower, i.e., aggression is higher.

Addressing the first question, we observed that on each of our visits, ravens and magpies began arriving at the CWWC prior to the beginning of the daily feeding tours and entered enclosures as the wolves were fed, indicating that food was their primary incentive. At other times of the day, there were few to no corvids around wolf enclosures. The Mexican gray wolf enclosure 7 attracted the highest number of ravens across most visits; and the single wolf in enclosure 8—the oldest wolf at the CWWC—attracted the fewest ravens. Enclosure 8, the last enclosure on the feeding tour, was relatively isolated from the other wolf enclosures, which may have reduced visitation by corvids (Fig 1). Smaller numbers of magpies than ravens in general visited the CWWC, with only one or two per enclosure at most, so our sample sizes were lower. No magpies visited enclosure 8.

As noted above, the Mexican gray wolf enclosure consistently had the highest number of ravens observed compared to the other enclosures, across most dates. The Mexican gray wolf enclosure had to meet the requirements of the USFWS for an endangered species and differed from other enclosures. The fence is made of chain link 9-gauge wire and stands 2.44 m high, with nearly a meter of fence cantilevered at the top. Furthermore, the Mexican gray wolves have less interaction with humans than do the other gray wolves. According to the CWWC staff, the Mexican gray wolves behave differently than the gray wolves. These factors—either enclosure construction or differences in wolf behavior—may be more conducive to raven visitation.

We found that many ravens and magpies did obtain food from wolf enclosures. On average, 0.32 ±  0.35 of the ravens and 0.37 ±  0.41 magpies per enclosure obtained food from wolves, but the proportion rewarded varied by enclosure (Table 2). Cumulatively, overall 0.33 of ravens (95% CI =  0.28 to 0.36) and 0.43 of magpies (95% CI =  0.31 to 0.56) per enclosure obtained some amount of food, which may represent a higher success rate than experienced with some prey items in the wild and an incentive for visiting wolf enclosures. In addition, the food reward—i.e., pieces of meat—could be considered high quality (Table 5).

Individual wolves reacted somewhat differently to the presence of the birds. The highest proportion of ravens obtained food in enclosure 8, but the number of ravens visiting that enclosure was low. The lowest proportion of ravens that obtained food was in enclosure 4. During our observations, the wolf pair in this enclosure always consumed their food quickly, making it difficult for the birds to get any scraps. For instance, wolf 4-2 is known to promptly take its food to a tree stump, place it on the stump, and then feed. Magpies in enclosure 7 obtained the highest proportion of food rewards, and magpies in enclosure 2 the lowest proportion.

Given that individual ravens and magpies were not marked and could not be followed during each feeding tour, it is possible that individuals that did not obtain food in some enclosures did obtain food in other enclosures. In other words, the daily proportions of ravens and magpies that obtained food from wolf enclosures could be underestimated in our study. In order to track the feeding success of individual corvids, a number of them would need to be distinctively marked—for example with colored leg bands.

Our observations indicated that landing in wolf enclosures and attempting to obtain the wolves’ food during feeding posed relatively low risk of harm or death to corvids. Of the total number of potential raven-wolf interactions (n =  450), ravens were chased in 6 or only 1.3% of interactions. Of the 65 potential magpie-wolf interactions, only 2 birds were chased, representing only about 3% of interactions. We assume that, normally, ravens and magpies are out foraging on their own most of the day and show up at CWWC around feeding time because of the high likelihood of receiving high quality food with low individual risk. Reports from the staff that a raven and a magpie were recently killed by wolves at the CWWC, however, reinforce the fact that risk exists.

The number of ravens and magpies visiting the CWWC varied throughout the study. We found that raven numbers were negatively influenced by daily maximum and average temperature (Table 3). This suggests that ravens were more inclined to visit wolves for food when temperatures were lower, indicating some energetic (caloric) benefits from the additional food, which may help individuals endure cold nights. In contrast, magpie numbers were positively influenced by daily minimum and average temperatures, suggesting that warmer temperatures led to higher visitation. The explanation for differences in the relationship between temperature and numbers of ravens and magpies is not clear; however, magpies are frequent food cachers [12,13] and may rely on food caches rather than opportunistic foraging when temperatures drop. The seasonal decline in numbers of magpies visiting the CWWC may have been influenced by breeding activities. The black-billed magpie breeding season begins in March and may continue through June [12]. Strong trends occurred between food amount provided per day and proportion of ravens that obtained food, but this relationship did not hold for magpies (Table 4).

Given the daily presence of corvids during feeding and potential loss of food to wolves, we ask why wolves exhibit a relatively high level of tolerance, especially towards ravens. This tolerance is also exhibited by wolves in the wild. One explanation given for the high tolerance by wolves is simply the high energetic costs of constantly defending their food against losses to corvids (kleptoparasitism), as suggested by [15]. Alternatively, wolf tolerance could be an innate response evolved over millennia, derived from an historically beneficial association between wolves and ravens, which is described in indigenous stories, TEK, and observed in the wild, such as ravens leading wolves to potential prey or carcasses (e.g., [2,14]). Magpies, however, may be primarily opportunistic foragers where they are sympatric with wolves; in other words, they do not appear to interact with wolves in the wild in the mutually beneficial ways attributed to ravens. They are present in lower numbers than ravens both at the CWWC and at wolf kills in the wild and may more easily escape wolf attention [16,17].

In order to determine whether the tolerance shown by wolves to corvids may be unique, we observed the reactions of two other canids at the CWWC to ravens and magpies that attempted to steal their food. The coyotes at CWWC were aggressive toward ravens that entered their enclosures. For example, we witnessed coyotes chasing a raven which was approaching a piece of meat; the coyotes literally leaped into the air attempting to catch the raven. Stahler et al. [14] provide data from Yellowstone National Park which indicated that ravens infrequently associated with coyotes, in contrast to wolves. The two New Guinea singing dogs at the CWWC were even more aggressive towards ravens and magpies, and staff members report that the dogs have killed several birds. Currently, the New Guinea singing dogs are only rarely visited by ravens or magpies. The New Guinea singing dogs and the coyotes are usually fed about 30 minutes before the wolves, which would attract visiting corvids. The high risk of injury may deter the corvids from entering their enclosures.

Tolerance of corvids may occur in other wolf species as well. At the CWWC, a red wolf was lying on the ground, chewing on a bone from a previous feeding, when a magpie approached the wolf and began pecking at the wolf’s rump. The magpie continued this behavior for about 8 minutes, eliciting no reaction from the wolf. The magpie finally stopped and walked over to the tip of the wolf’s tail, lifted the tail up with its beak and moved the tail, again with no response. A minute later, a raven joined the magpie and began to peck at the wolf’s tail, while the magpie watched. The raven left after a minute of pecking. Next, the same magpie was joined by another magpie and both began to peck at the wolf’s rump once again. Finally, the wolf stood up and walked away, revealing a meat scrap that it had been lying on, which was obviously known by the corvids. A raven and a magpie promptly began feeding on the meat.

## Concluding comments

Of the ravens and magpies that visit the CWWC and enter wolf enclosures during wolf feeding, an estimated 33% and 43%, respectively, obtain food, with a comparatively low risk of harm. The birds were unmarked, so that individuals that did not obtain food in one enclosure may have obtained food in other enclosures. In other words, these percentages could be underestimates of overall feeding success.

Higher numbers of ravens visited enclosures on days with lower daily maximum and lower daily average temperatures, whereas more magpies occurred on days with higher daily low and higher daily average temperatures. Food type did not affect corvid numbers, but a trend existed between the proportion of ravens that obtained food and food amount provided to wolves.

The likelihood of obtaining food may explain why ravens, and to a lesser extent magpies, appear most days in time for the wolf feeding tours. However, very few ravens and magpies visited the coyotes and New Guinea singing dogs at the CWWC, most likely because these canids show lower tolerance for losing their food to the corvids and are more aggressive than the gray wolves, increasing the level of risk.

The interaction at CWWC between corvids and wolves appears to parallel the interaction between corvids and gray wolves in the wild, where ravens, and sometimes magpies, show up at wolf kills and often feed in proximity to wolves [14,16,17]. Ravens have been documented taking, collectively, large proportions of wolf kills [21,22]. The simplest explanation for the presence of ravens and magpies in wolf enclosures at the CWWC is that corvids, and ravens especially, are highly opportunistic and calculating foragers (e.g., [2]), and that food provided gray wolves (and other animals) in captivity is a foraging opportunity for corvids. This behavior is unlikely to result from historical cultural transmission among corvids, given that wolves were extirpated in Colorado by the 1940s [24], although there are recent natural and on-going agency-managed reintroductions of wolves in the state [29].

Tolerance by gray wolves for food loss to corvids at the CWWC is more challenging to explain. Explanations for tolerance of gray wolves towards ravens and magpies may be the high energetic cost of chasing off birds so often (e.g., [15]) or tolerance may have evolved over millennia from some mutual benefit. Regarding the latter explanation, Heinrich [2] notes that the relationship between ravens and wolves may reflect “an ancient evolutionary history” rooted in mutualistic interaction. Further studies would be valuable to determine whether the wolf-raven relationship has in fact been shaped by coevolutionary interaction. Whether the relationship between ravens and wolves is coevolved or culturally learned by ravens and magpies, the interactions provide opportunities at wolf sanctuaries for natural history interpretation and education for visitors.

## Supporting information

S1 Text M. W. Price. 2022. Personal communication.
(DOCX)

S2 Text S. Coldiron. 2023. Personal communication.
(DOCX)

S1 File Data dictionary and database for CWWC wolf-corvid interactions study. (XLSX)

## References

[1] Pika S, Sima MJ, Blum CR, Herrmann E, Mundry R. Ravens parallel great apes in physical and social cognitive skills. Sci Rep. 2020;10:20617. doi: 10.1038/s41598-020-77060-8 33303790 PMC7728792

[2] Heinrich B. Mind of the Raven: Investigations and Adventures with Wolf-Birds. New York: HarperCollins Publishers; 1999.

[3] Marzluff JM, Angell T. In the Company of Crows and Ravens. Ukraine: Yale University Press; 2005.

[4] Boeckle M, Schiestl M, Frohnwieser A, Gruber R, Miller R, Suddendorf T, et al. New Caledonian crows plan for specific future tool use. Proc Royal Soc B Biol Sci. 2020;287(1938):20201490. doi: 10.1098/rspb.2020.1490 PMC7735258 33143583

[5] Van Horik JO, Clayton NS, Emery NJ. Convergent evolution of cognition in corvids, apes and other animals. In: The Oxford Handbook of Comparative Evolutionary Psychology. Oxford University Press; 2012; p. 80–101.

[6] Kamil AC, Balda RP, Olson DJ. Performance of four seed-caching corvid species in the radial-arm maze analog. J Comp Psychol. 1994;108: 385–393. 7813195 10.1037/0735-7036.108.4.385

[7] Tomback DF. How nutcrackers find their seed stores. Condor. 1980;82:10–19.

[8] Vander Wall SB. An experimental analysis of cache recovery in Clark’s nutcracker. Anim Behav. 1982;30:84–94.

[9] Seed A, Emery N, Clayton N. Intelligence in corvids and apes: a case of convergent evolution? Ethol. 2009;115:401–420. doi: 10.1111/j.1439-0310.2009.01644.x

[10] Kaplan G. Play behaviour, not tool using, relates to brain mass in a sample of birds. Sci Rep. 2020;10:20437. doi: 10.1038/s41598-020-76572-7 33235248 PMC7687885

[11] Boarman Wl, Heinrich B. Common Raven (Corvus corax), version 1.0. In: Birds of the World (Billerman SM, Editor). Cornell Lab of Ornithology. Ithaca (NY); 2020. doi: 10.2173/bow.comrav.01

[12] Trost CH. Black-billed Magpie (Pica hudsonia), version 1.0. In: Billerman SM, ed. Birds of the World. Cornell Lab of Ornithology; 2020. doi: 10.2173/bow.bkbmag1.01

[13] Birkhead TR. The Magpies: the Ecology and Behaviour of Black-billed and Yellow-billed Magpies. London: T. & A. D. Poyser; 1991. p. 270.

[14] Stahler D, Heinrich B, Smith D. Common ravens, Corvus corax, preferentially associate with gray wolves, Canis lupus, as a foraging strategy in winter. Anim Behav. 2002;64(2). doi: 10.1006/anbe.2002.3047

[15] Vucetich JA, Peterson RO, Waite TA. Raven scavenging favors group foraging in wolves. Anim Behav. 2004;67(6). doi: 10.1016/j.anbehav.2003.06.018

[16] Smith DW. Ten years of Yellowstone wolves. Yellowstone Science. 2005;13(1):1–48.

[17] Smith DW, Peterson RO, Houston DB. Yellowstone after wolves. BioSci. 2003;53:330–340.

[18] Selva N, Jedrzejewska B, Jedrzejewski W. Scavenging on European bison carcasses in Bialowieza Primeval Forest (eastern Poland). Ecoscience. 2003;10:303–311.

[19] Jessen TD, Ban NC, Claxton N, Darimont CT. Contributions of Indigenous Knowledge to ecological and evolutionary understanding. Front Ecol Environ. 2022;20(2). doi: 10.1002/fee.2435

[20] Suiseeya KRM, O’Connell MG, Leoso E, Defoe MSBN, Anderson A, Bang M, et al. Waking from paralysis: revitalizing conceptions of climate knowledge and justice for more effective climate action. Ann Am Acad Pol Soc Sci. 2022;700:166–182. doi: 10.1177/00027162221095495

[21] Ballard WB, Ludwig NB, Carbyn NW, Smith DW. Wolf interactions with non-prey. In: Mech LD, Boitani L, editors. Wolves: Behavior, Ecology, and Conservation. Chicago and London: University of Chicago Press; 2003: p. 259–271.

[22] Hayes RD, Baer AM, Wotschikowsky U, Harestad AS. Kill rate by wolves on moose in the Yukon. Can J Zool. 2000;78(1):49–59. doi: 10.1139/z99-187

[23] Bugnyar T, Kotrschal K. Observational learning and the raiding of food caches in ravens, Corvus corax: Is it ‘tactical’ deception? Anim Behav. 2002;64(2):185–195. doi: 10.1006/anbe.2002.3056

[24] Phillips M, Bishop N, Gardner C. The wolf in Colorado. In: Carhart AH, Gulliford A, Wolf T, editors. The Last Stand of the Pack: Critical Edition. Colorado: University Press of Colorado; 2017. p. 251–280.

[25] Surbakti S, Parker HG, McIntyre JK, Maury HK, Cairns KM, Selvig M, et al. New Guinea highland wild dogs are the original New Guinea singing dogs. Proc Natl Acad Sci USA. 2020;117(39): p. 24369–24376. doi: 10.1073/pnas.2007242117 32868416 PMC7533868

[26] Wikenros C, Gicquel M, Zimmermann B, Flagstad Ø, Åkesson M. Age at first reproduction in wolves: different patterns of density dependence for females and males. Proc Roy Soc B. Biol Sci. 2021;288(1948):20210207. doi: 10.1098/rspb.2021.0207 PMC8059544 33823674

[27] Hilbe JM. Modeling Count Data. Cambridge University Press; 2014.

[28] Brooks ME, Kristensen K, van Benthem KJ, Magnusson A, Berg CW, Nielsen A, et al. glmmTMB balances speed and flexibility among packages for zero-inflated Generalized Linear Mixed Modeling. R J. 2017;9(2):378–400. doi: 10.32614/RJ-2017-066

[29] Colorado Parks and Wildlife. Colorado Wolf Management and Restoration Plan; 2023. p. 107.

