# Supplementary material for: Dining with wolves: Are the rewards worth the risks?
Source: PLoS One. 2025 Mar 24;20(3):e0319565. doi: 10.1371/journal.pone.0319565 (PMC11932469; doi:10.1371/journal.pone.0319565)
Supplement: S1 Text — 2022. Personal communication. (DOCX) [file pone.0319565.s001.docx]

**Supporting Information**

**S1 Text**, M. W. Price. 2022. Personal communication.

**From:** Michael Price <[migizee@yahoo.com](mailto:migizee@yahoo.com)>
**Sent:** Thursday, October 6, 2022 11:36 AM
**To:** Merrell, Summer <[SUMMER.MERRELL@UCDENVER.EDU](mailto:SUMMER.MERRELL@UCDENVER.EDU)>
**Subject:** Fw: History of Wolves and Ravens

Authors’ Note: The first author is Summer N. Richman (Merrell)

Thurs, 10/06/2022, 11:36am

Hello Summer,

Thank you for reaching out. And, thank you for your kind words about my presentation. Within our cultural stories, the relationship between the wolf and the raven are well known. They both work together to find food in the frozen north. The ravens are not strong enough to rip into the carcass of a dead animal, so they sit in the trees and call on wolves to come a rip up and feed on the carcass. The ravens sit in the trees patiently awaiting their turn. After the wolves are done feeding, there is still enough meat on the bones to feed the ravens. Science calls this symbiosis. We see it as a much deeper relationship between two spiritual beings. And, for us Anishinaabe, we learn lessons from these stories like cooperation, patience and mutual benefit. Our stories, ceremonies, songs, and traditions help us, as Anishinaabe people, to live a balanced and sustained life with all other beings. The animals and landscape become our teachers. We recollect our teachings every time we see a raven, or more rarely a wolf, in the forest.

As for Magpies, they are beautiful birds. I saw them all the time when I lived in Montana from 2008-2012. Here in Minnesota and Wisconsin, we never saw magpies. But, nowadays, I see them in northcentral Minnesota. They are apparently moving eastward. We do not have traditional stories about magpies because they are a newcomers to the Great Lakes region. I am sure many of the Plains tribes have stories about magpies.

I hope this helps. Let me know if you have any other questions. I would be glad to help.

Michael
