# Supplementary material for: Dining with wolves: Are the rewards worth the risks?
Source: PLoS One. 2025 Mar 24;20(3):e0319565. doi: 10.1371/journal.pone.0319565 (PMC11932469; doi:10.1371/journal.pone.0319565)
Supplement: S2 Text — 2023. Personal communication. (DOCX) [file pone.0319565.s002.docx]

**Supporting Information**

**S2 Text**, S. Coldiron. 2023. Personal communication.

Authors’ Note: Rist Canyon, northern Colorado, was the old location of the wolf sanctuary, which was recently moved to a more remote area.

Wed 11/22/2023 1:43 PM

Hi Diana:

We had ravens and magpies at Rist Canyon.  We just moved the last animals Sept. 29th.  We have magpies trying to steal the animals food.  No ravens yet.

We are still standing up our protocols at our new facility near Red Feather Lakes.   So let's revisit this late Spring after we go through our first winter and early spring.

You have a great Thanksgiving as well.

Shelley

Shelley J. Coldiron, PhD

Executive Director

W.O.L.F.
